# Ultrasmall Gold Nanoparticles Radiolabeled with Iodine-125 as Potential New Radiopharmaceutical

**DOI:** 10.1021/acsabm.3c01158

**Published:** 2024-02-07

**Authors:** Runze Wang, Huanhuan Liu, Bas Antal, Hubert Th. Wolterbeek, Antonia G. Denkova

**Affiliations:** † Applied Radiation and Isotopes, Department of Radiation Science and Technology, Faculty of Applied Sciences, Delft University of Technology, Mekelweg 15, 2629 JB Delft, The Netherlands; ‡ Department of Medical Imaging, Henan Provincial People’s Hospital & the People’s Hospital of Zhengzhou University, Zhengzhou 450003, P. R. China

**Keywords:** radionuclide therapy, Auger therapy, Auger electron, iodine-125, ultrasmall gold nanoparticle

## Abstract

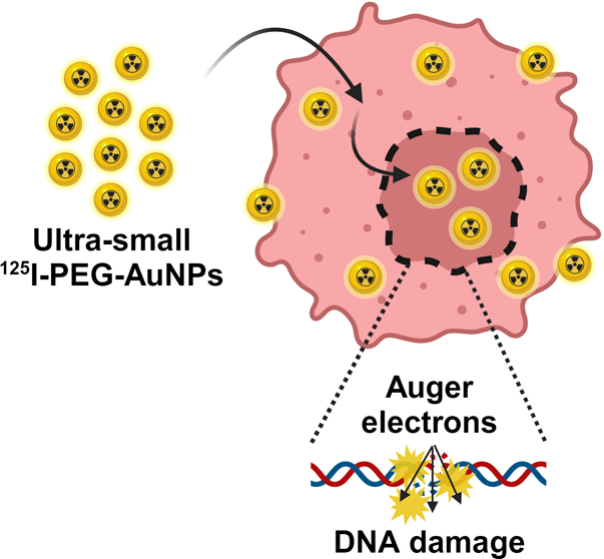

The relatively high linear energy transfer of Auger electrons, which can cause clustered DNA damage and hence efficient cell death, makes Auger emitters excellent candidates for attacking metastasized tumors. Moreover, gammas or positrons are usually emitted along with the Auger electrons, providing the possibility of theragnostic applications. Despite the promising properties of Auger electrons, only a few radiopharmaceuticals employing Auger emitters have been developed so far. This is most likely explained by the short ranges of these electrons, requiring the delivery of the Auger emitters to crucial cell parts such as the cell nucleus. In this work, we combined the Auger emitter ^125^I and ultrasmall gold nanoparticles to prepare a novel radiopharmaceutical. The ^125^I labeled gold nanoparticles were shown to accumulate at the cell nucleus, leading to a high tumor-killing efficiency in both 2D and 3D tumor cell models. The results from this work indicate that ultrasmall nanoparticles, which passively accumulate at the cell nucleus, have the potential to be applied in targeted radionuclide therapy. Even better tumor-killing efficiency can be expected if tumor-targeting moieties are conjugated to the nanoparticles.

## 1 Introduction

Radionuclide therapy (RNT) is a cancer treatment modality that uses internal radiation to primarily attack cancer metastases. Targeting agents such as antibodies and peptides are typically coupled via bifunctional chelators to radionuclides emitting α particles, β^–^ particles, or Auger electrons (AE).^1^ The emitted particles can damage DNA molecules of cancer cells either directly or indirectly, eventually leading to the death of the tumor cells.^2^ Numerous preclinical and clinical trials have been carried out to verify the clinical potential of α and β^–^ emitters based radiopharmaceuticals in the past years.^3−7^ However, studies on applying AE emitters for cancer treatment are still limited.

AEs are commonly emitted by radionuclides that decay via electron capture (EC) or internal conversion (IC). The AEs have energy from 10 eV to 10 keV but with a very short-range of only a few nanometers, resulting in intermediate linear energy transfer (LET) from 4 to 26 keV/μm. Due to the short-range of AEs, they must be emitted close enough to the DNA strands to produce dense ionizations and excitations.^8^ Thus, the AE emitters must be targeted to the cell nucleus or other crucial cell organelles to achieve optimal tumor-killing efficiency.^9^ High nucleus uptake of AE emitters has been previously achieved by radiolabeling AE emitters on nucleosides or tumor-targeting peptides containing nuclear localization sequence (NLS).^10−14^

Besides nucleosides and antibodies, nanoparticles have also been found to accumulate at the cell nucleus actively or passively.^15,16^ As reported previously, spherical nanoparticles with a size of 9 nm or less are able to cross the nuclear pore complex by diffusion.^17^ For larger nanoparticles, surface modification by peptides with NLS is always required to achieve high accumulation at the cell nucleus.^18,19^ For instance, 10 to 30 nm gold nanoparticles (AuNPs) modified with epidermal growth factor (EGF) or trastuzumab have been radiolabeled with ^111^In and have shown high tumor-killing efficiency due to the enhanced localization at the cell nucleus.^20−22^ However, the high liver and spleen uptake of these large nanoparticles due to the mononuclear phagocyte system (MPS) capture limits their tumor uptake and might lead to radiation burden to the liver and spleen.^23^

Iodine-125 (^125^I) is a typical AE emitter having 24.9 AEs emitted per decay and a half-life of 59.4 days, which is widely used therapeutically in the brachytherapy for brain tumors, prostate cancer as well as head and neck cancer.^24−30^ Besides brachytherapy, other clinical application of ^125^I in RNT is very rare, and the results are rather disappointing. In early studies, primarily nucleoside 5-iodo-2-deoxyuridine (^125^I-UdR) has been used, which showed only tiny tumor accumulation due to metabolic breakdown of the ^125^I-UdR. These findings have led to direct tumor injection of the radiopharmaceutical rather than systemic treatment, which, unfortunately, does not help in attacking metastasized cancer.^31−33^ Preclinically, a number of studies do show that ^125^I has good therapeutic potential provided that sufficient tumor accumulation is achieved.^34−37^ Instead of using small organic molecules to carry ^125^I that are prone to metabolic breakdown, we suggest applying ultrasmall gold nanoparticles as the carrier for ^125^I. Such nanoparticles radiolabeled with ^125^I have already been reported in positron emission tomography (PET) imaging studies, showing the fast tumor targeting and high radiochemical stability *in vivo*.^38,39^ However, few studies on the therapeutic effect of ^125^I radiolabeled nanoparticles have been reported so far.^40^

In this work, we investigated the potential of polyethylene-glycol (PEG)-coated AuNPs with a size of just 2 nm, radiolabeled with ^125^I through a simple and fast method achieving high radiolabeling efficiency and high radiochemical stability. The uptake, subcellular distribution, and tumor-killing efficiency of the ^125^I-PEG-AuNPs were studied *in vitro* using 2D monolayer or 3D tumor spheroid cell models revealing high tumor-killing efficiency.

## 2 Methods and Materials

### 2.1 Materials

Gold(III) chloride trihydrate (≥99.9%, HAuCl_4_·3H_2_O) and crystal violet (1% solution) were purchased from Merck Sigma (Zwijndrecht, The Netherlands). PEG-SH (Mw = 750 Da) was obtained from Rapp Polymere (Tuebingen, Germany). Iodine-125 (17 mCi/mg, pH 12–14, ^125^I) was supplied by PerkinElmer. All chemicals were used as received without further purification. MiliQ water was obtained from an in-house MiliQ system (Millipore) and was used throughout this study.

### 2.2 Synthesis of 2 nm PEG-AuNPs

The synthesis of PEG-AuNPs was adapted from a published method with minor adjustments.^41^ In a typical synthesis, 25 mL of 2.4 mM PEG750-SH water solution was mixed with 75 μL of 1 M HAuCl_4_ at room temperature for 30 min. The mixture was then heated at 95 °C for 35 min under vigorous stirring. The resulting PEG-AuNPs were collected and filtered by a 220 nm syringe filter to remove the large aggregates, followed by thrice water washing using centrifuge filters (Amicon, MWCO 10,000) to remove free small ligands. The final volume was adjusted to 2 mL after an extra wash by PBS buffer (pH 7.4) and stored at 4 °C.

### 2.3 Characterization of PEG-AuNPs

The shape and size of the PEG-AuNPs were imaged with a 120 kV JEM-1400 Plus transmission electron microscope (TEM, JEOL). The size distribution of the nanoparticles was studied by measuring the size of at least 150 particles in each sample. The absorption spectrum of PEG-AuNPs was measured by a UV–Vis–NIR spectrophotometer (UV-6300PC, VWR). The hydrodynamic diameter and ζ potential of the PEG-AuNPs were determined by a Zetasizer (nano-ZS, Malvern). To determine the concentration of gold content in each sample, 10 μL of a sample was first dissolved in 100 μL of aqua regia and then measured using ICP-OES (Optima 8000, PerkinElmer).

### 2.4 Radiolabeling of ^125^I on PEG-AuNPs

The ^125^I stock solution was neutralized by the same volume of 0.1 M HCl right before being added to the PEG-AuNPs. In typical sample preparation, 37 MBq ^125^I was added to 100 μL 46 μM PEG-AuNPs (^125^I:NP = 0.1) and shaken at 600 rpm for 30 min at 37 °C. The radiolabeling efficiency was monitored by iTLC (mobile phase/acetonitrile/water = 1:3). The ^125^I-PEG-AuNPs remained at the origin while the free ^125^I was located at the top of the strip. The iTLC strips were dried in air and then exposed to a phosphor screen for 15 min. The phosphor screen was scanned using a Typhoon Trio phosphorimager (GE Healthcare). The obtained images were analyzed by using ImageQuant TL software (GE Healthcare) to calculate the radiolabeling efficiency. A typical iTLC radiochromatogram of the ^125^I-PEG-AuNPs right after the radiolabeling is shown in Figure S4.

### 2.5 *In Vitro* Colloidal Stability

The PEG-AuNPs were dispersed in PBS and 10% fetal bovine serum (FBS) and incubated at 37 °C for 72 h. The UV–Vis spectrum of each PEG-AuNP dispersion was measured every 24 h. Besides, the hydrodynamic diameter of PEG-AuNPs in PBS and MiliQ water was also measured every 24 h.

### 2.6 *In Vitro* Radiochemical Stability

The ^125^I-PEG-AuNPs were incubated in PBS and 10% FBS at 37 °C for 72 h. The release of ^125^I from PEG-AuNPs was evaluated by iTLC every 24 h using the same mobile phase as described in Section 3.

### 2.7 Cell Culture

The U87 human glioblastoma cells were obtained from ATCC and cultured in complete Dulbecco’s modified Eagle’s medium (DMEM) supplemented with 10% FBS and 1% penicillin/streptomycin in a cell incubator (Heracell, Heraeus), providing a humidified atmosphere containing 5% CO_2_ at 37 °C.

### 2.8 *In Vitro* Cell Viability Assay

U87 cells were plated on 96-well plates with a cell density of 5000 cells/well. After preincubation for 24 h, the culture medium was replaced by fresh culture medium containing 1 to 1000 nM PEG-AuNPs and incubated for another 24 h. The cells unexposed to PEG-AuNPs were used as control. The cells were then washed twice with PBS and fed with fresh culture medium containing a 10% cell counting kit-8 (CCK-8, Dojindo Laboratories). The absorbance of the cells at 450 nm was measured by a microplate scanning spectrophotometer (PowerWave XS, Bio-Tek) after incubating the cells at 37 °C for another 1 to 2 h. The relative viability of each group was then calculated by comparing the absorption at 450 nm with that of the control group.

### 2.9 Uptake and Subcellular Distribution of ^125^I-PEG-AuNPs in Monolayer Cells

#### 2.9.1 Total Uptake

U87 cells were plated on 12-well plates and preincubated for 24 h (8 × 10^4^ cells/well). On the next day, 1 mL of fresh culture medium containing 10, 50, and 100 nM ^125^I-PEG-AuNPs (37 kBq) was added to the cells. The cells were then incubated for another 4 or 24 h at 37 °C. After incubation, the cells were washed three times with PBS to remove free PEG-AuNPs and completely lysed with 0.1 M NaOH. The wash fractions and the lysed cell fractions were finally counted in an automated γ counter (Wallac Wizard^2^ 2480, PerkinElmer). The calculations of the number of ^125^I-PEG-AuNPs internalized per cell is explained in the Supporting Information.

#### 2.9.2 Subcellular Distribution

To determine the subcellular distribution of ^125^I-PEG-AuNPs in monolayer cells, the cells were thoroughly washed after being incubated with different concentrations of ^125^I-PEG-AuNPs for 4 or 24 h and then detached by trypsin. The subcellular fractions of cells were separated using the Subcellular Protein Fractionation Kit (Thermo Scientific) following the instructions of the manufacturer. The counts of each cell fraction were measured by an automated γ counter (Wallac Wizard^2^ 2480, PerkinElmer).

#### 2.9.3 Silver Staining

U87 cells were seeded on 6-well plates with a cell density of 8 × 10^4^ cells/well and preincubated for 24 h. On the next day, the cells were treated by 1, 50, and 100 nM PEG-AuNPs for another 24 h followed by thrice PBS wash. Silver staining of the cells was performed using the LI silver enhancers kit (Nanoprobe) following the instructions from the manufacturer. The stained cells were imaged using an inverted light microscope (AE2000, Motic).

### 2.10 Uptake of ^125^I-PEG-AuNP in Cell Spheroids

U87 cells were seeded on U-shaped 96-well plates and preincubated for 7 days (2000 cells/well) to form the spheroids. After the formation of spheroids, 200 μL of culture medium containing 10, 50, or 100 nM ^125^I-PEG-AuNPs (37 kBq) was added to the spheroids and incubated for 4 or 24 h at 37 °C. The spheroids were then washed three times by PBS before measuring the counts of the wash and the spheroid fractions using an automated γ counter (Wallac Wizard^2^ 2480, PerkinElmer).

### 2.11 *In Vitro* Cytotoxicity of ^125^I-PEG-AuNPs in Monolayer Cells

#### 2.11.1 Viability Assay

U87 cells were seeded on 96-well plates with the cell density of 5000 cells/well and preincubated at 37 °C for 24 h. After preincubation, the culture medium was removed, and 100 μL of fresh culture medium containing ^125^I-PEG-AuNPs with 37, 370, or 740 kBq of ^125^I was added and incubated at 37 °C for 24 h. The cells exposed to culture medium and cells exposed to 740 kBq [^125^I]NaI were used as control. On the next day, the cells were washed three times by PBS, fed with 100 μL fresh culture medium, and incubated at 37 °C for another 24 h before the cell viability of each group was measured by the CCK-8 assay. (*n* = 4)

#### 2.11.2 DNA Proliferation Assay

After the CCK-8 assay, the cells were washed twice by PBS and 200 μL of water was added to each well. The cells were, hereby, repetitively frozen (−20 °C) and thawed (37 °C) for membrane destruction. Then, the DNA content of each well was measured using the AccuClear dsDNA quantification kit (Biotum) following the instructions from the manufacturer. In brief, 50 μL of each sample was added to 200 μL of working solution prepared by diluting the dye 100 times with the DNA quantification buffer and mixed by pipetting. After incubating at room temperature for 5 min in the dark, the plate was read on a fluorescent spectrometer with excitation and emission settings of 468 and 507 nm, respectively. The obtained results were fitted to a DNA standard curve to determine the mass of DNA per sample and normalized to the control group to determine the proliferation capacity (*n* = 4).

#### 2.11.3 Colony Formation Assay

U87 cells were seeded on 12-well plates with a cell density of 8 × 10^4^ cells/well and preincubated at 37 °C for 24 h. Then, the cells were incubated with ^125^I-PEG-AuNPs containing 0.37, 0.74, 1, or 3.7 MBq of ^125^I or 3.7 MBq of [^125^I]NaI for another 24 h. The cells exposed to culture medium were used as control. The next day, the cells were washed three times by PBS to remove free activity and reseeded in 6-well plates with a cell density of 500 cells/well. The cells were then left undisturbed for 14 days to allow colony formation. The culture medium was refreshed every 3 days. On the last day, the colonies were fixed by 4% (w/v) paraformaldehyde, stained by 1% crystal violet and counted manually on an inverted light microscope (Gapelcom, *n* = 3).

### 2.12 *In Vitro* Cytotoxicity of ^125^I-PEG-AuNPs in 3D Cell Spheroids

#### 2.12.1 Spheroid Growth Inhibition Assay

U87 cells were seeded in a U-shaped 96-well plate at the cell density of 2000 cells/well and incubated for 7 days to form spheroids. After the formation of spheroids, the culture medium containing ^125^I-PEG-AuNPs (37, 370, and 740 kBq ^125^I) or 740 kBq [^125^I]NaI was added and incubated at 37 °C. After being incubated for 24 h, the spheroids were washed three times by PBS and fed with fresh medium. The growth of the spheroids was followed in a time of up to 13 days by capturing images using an inverted light microscope (Gapelcom). The size of the spheroids was analyzed with ImageJ. Nontreated spheroids were used as a control (*n* = 4)

### 2.13 Absorbed Dose Calculation

The dose calculations were performed using the Monte Carlo toolkit TOPAS based on the Geant4 Simulation Toolkit.^42^ The simulation was based on the uptake and subcellular distribution of 50 or 100 nM ^125^I-PEG-AuNPs in 2D U87 cells after 24 h incubation (Table S1). The emission spectra of ^125^I obtained from the database MIRD (medical internal radiation dose) was used in this work.^43^ A nucleus radius of 5 μm and cell radius of 7 μm were used for the simulation of a single U87 cell.^44^ N.B. only the dose after removing excess activity (i.e., after first 24 h incubation) was calculated, since internalization data at early time points was not available. Moreover, the following two scenarios were applied in the dose calculation: (1) ^125^I was homogeneously distributed inside the cell nucleus while the Au content was simulated as a single particle in the middle of the cell nucleus; (2) ^125^I and Au were homogeneously distributed on the cell membrane and in the cytoplasm.

### 2.14 Statistical Analysis

Data are expressed as mean ± standard deviation based on at least three independent replicates. Student’s *t* test was used for the comparison between two samples. For the comparison among multiple samples, one-way or two-way ANOVA test was performed. *P* values: ns, *p* > 0.05, *p ≤ 0.05, **p ≤ 0.01, ***p ≤ 0.001, ****p ≤ 0.0001.

## 3 Results and Discussion

The application of nanomaterials in cancer treatment and diagnosis has been extensively reported.^45,46^ However, the high off-target uptake of nanoparticles in liver and spleen has raised the concern of long-term toxicity to healthy tissues.^23^ Nanoparticles with a hydrodynamic diameter less than 5.5 nm appear to be able to escape the MPS capture and to be rapidly excreted via the urinary system.^47,48^ In addition, such small nanoparticles have been found to pass through the nuclear pore complex (NPC) and accumulate in the cell nucleus.^49,50^ In this section, we combined 2 nm sized nanoparticles with ^125^I to develop a potential AE radiopharmaceutical.

**Figure 1:**
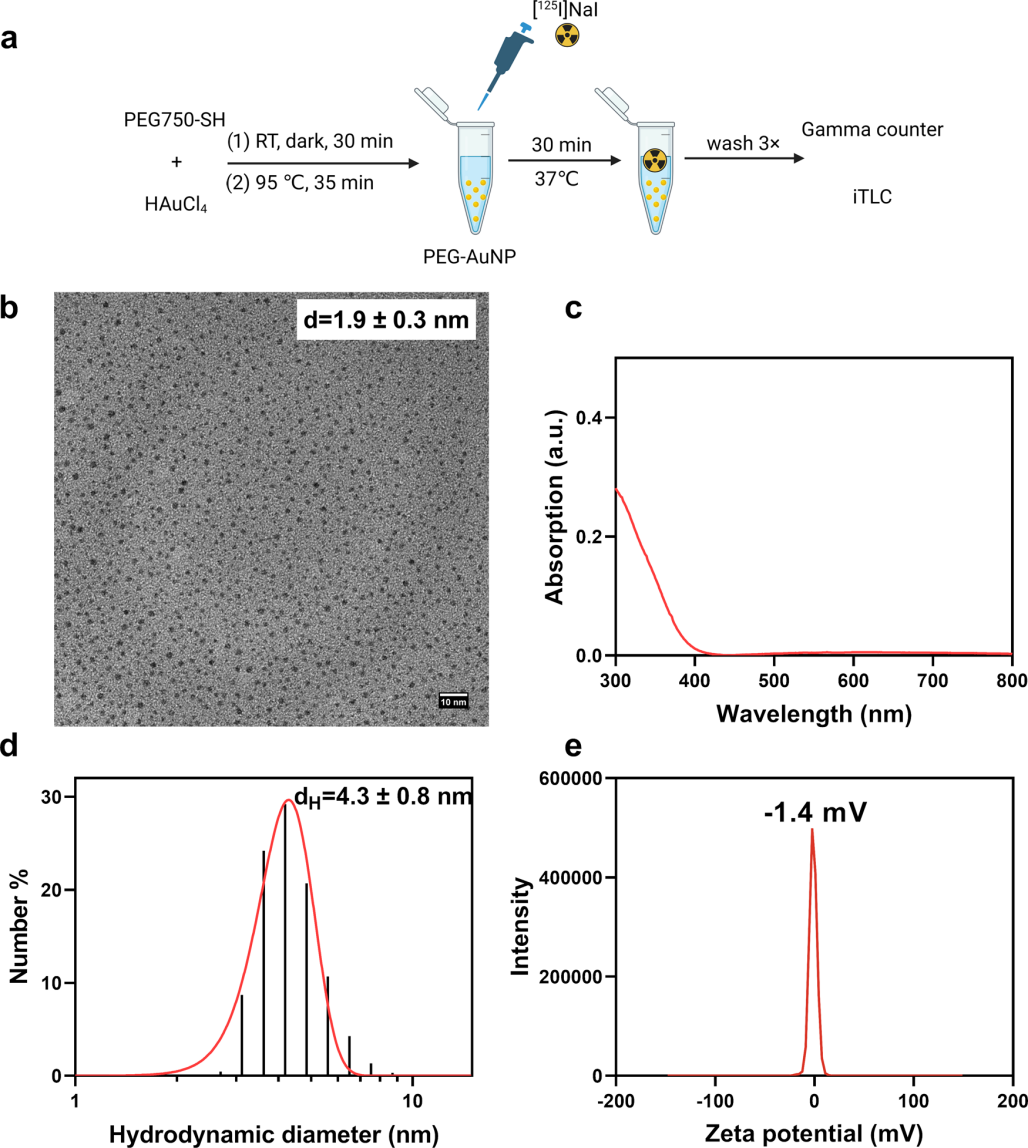
Synthesis and characterization of PEG-AuNPs: (a) schematic illustration of the synthesis and radiolabeling of PEG-AuNPs with ^125^I; (b) TEM image; (c) UV–Vis spectrum; (d) number weighted hydrodynamic diameter; and (e) ζ potential of PEG-AuNPs dispersed in PBS. Scale bar = 10 nm.

The ultrasmall PEG-AuNPs were first synthesized by the thermal reduction of HAuCl_4_ in the presence of PEG750-SH (Figure 1a).^41^ The core size of the PEG-AuNPs was determined by TEM imaging and appeared to be 1.9 ± 0.3 nm (Figure 1b). The small size of the PEG-AuNPs was further proven by the recorded UV–Vis spectrum where no obvious peak around 500 nm was observed (Figure 1c). As shown in Figure 1d, the hydrodynamic diameter of the PEG-AuNPs was measured by dynamic light scattering (DLS) and appeared to be 4.3 ± 0.8 nm. Furthermore, the PEG-AuNPs had neutral ζ potential, which can be ascribed to the PEG coating (Figure 1e).

**Figure 2:**
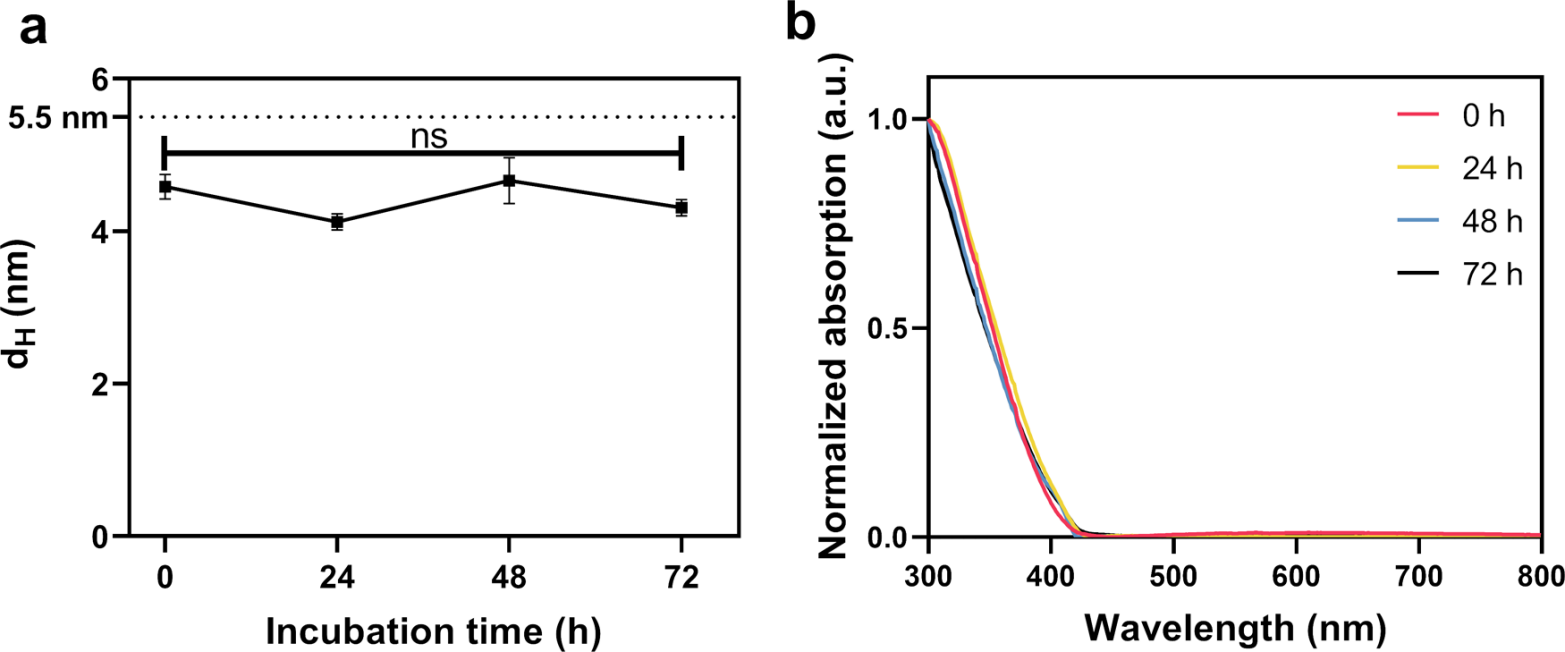
Colloidal stability of PEG-AuNPs: (a) the number weighted hydrodynamic diameter of PEG-AuNPs in PBS at 37 °C as a function of time, *n* = 3; (b) Normalized UV–Vis spectrum of PEG-AuNPs in 10% FBS at 37 °C and at different time points. *P* values: ns, *p* > 0.05.

Considering the complex environment in blood, the intravenously injected nanoparticles must maintain colloidal stability under similar conditions, such as when dispersed in physiological solutions. The obtained PEG-AuNPs were, therefore, dispersed in PBS or 10% FBS in PBS and incubated at 37 °C for 72 h. As shown in Figure 2a, the hydrodynamic diameter of the PEG-AuNPs in PBS was measured every 24 h and found to be unchanged for at least 72 h. Furthermore, no pronounced change in the UV–Vis spectrum of PEG-AuNPs in PBS (Figure S1) and 10% FBS (Figure 2b) was detected during 72 h incubation, indicating the high colloidal stability of PEG-AuNPs even in the presence of serum proteins.

**Figure 3:**
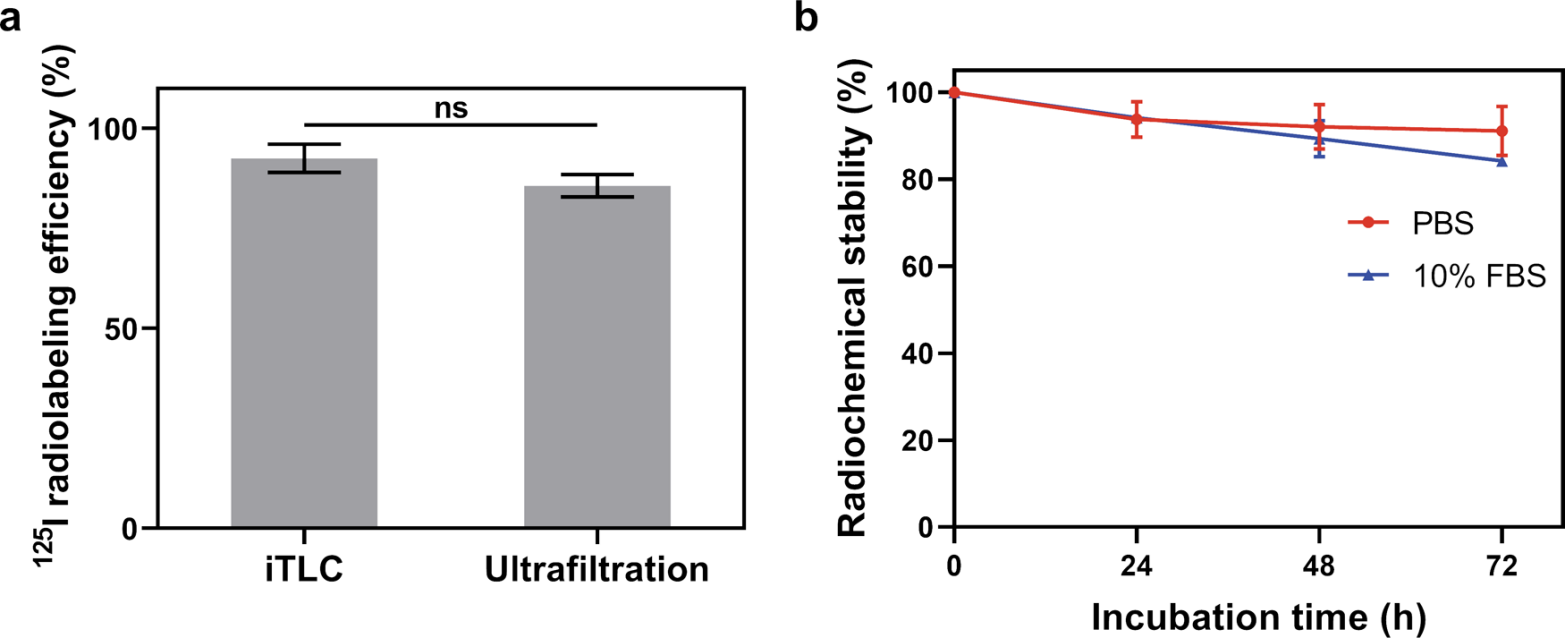
(a) Radiolabeling efficiency of ^125^I-PEG-AuNPs determined by iTLC and ultrafiltration experiments. ^125^I:NP = 0.1, [^125^I]NaI was neutralized before usage, *n* = 3; (b) radiochemical stability of ^125^I-PEG-AuNPs in PBS or 10% FBS over 72 h at 37 °C, *n* = 3. *P* values: ns, *p* > 0.05.

After confirmation of the small size and high colloidal stability of the PEG-AuNPs, radiolabeling with ^125^I was performed. As a soft base and acid, respectively, I^–^ ions have strong affinity to Au^0^.^51^ Thus, the radiolabeling of the PEG-AuNPs with ^125^I was utilized by chemisorption of ^125^I on the surface of the particles through the formation of Au–I bond.^38,52^ The radiolabeling conditions were optimized by using various molar ratios of the PEG-AuNPs and ^125^I^–^ ions and the pH values of the [^125^I]NaI solution. It was found that neutralizing the [^125^I]NaI solution to pH 7 using 0.1 M HCl before adding it to the PEG-AuNPs could significantly increase the radiolabeling efficiency, i.e., from ∼65% to more than 90% (Figure S2). We then studied the influence of the ^125^I to the NP ratio. As shown in Figures 3a and S3, a higher radiolabeling efficiency could be achieved by lowering the ^125^I to NP ratio. The radiolabeling efficiency was determined to be ∼100, 92.5, and 36% by iTLC when there was 2500×, 10×, and 2.5× excess of PEG-AuNPs, i.e., ^125^I:NP = 0.0004, 0.1, and 0.4, respectively. Increasing the number of PEG-AuNPs could boost the reaction between ^125^I^–^ and PEG-AuNPs by providing a larger surface area for reaction, thereby leading to higher radiolabeling efficiency. A ratio of ^125^I:NP of 0.1 was chosen for all of the following experiments in order to ensure high enough specific activity. After removing nonbound ^125^I with centrifuge filters, the ^125^I-PEG-AuNPs and washing solutions were counted using an automated γ counter for the determination of radiolabeling efficiency. A slightly lower radiolabeling efficiency of 85.7% was calculated based on these measurements versus iTLC, probably because of the removal of loosely bound ^125^I from the PEG-AuNPs (Figure 3a).

As free I^–^ could naturally accumulate in the thyroid, the high radiochemical stability of the ^125^I-PEG-AuNPs is critical for further *in vivo* applications.^53^ As shown in Figure 3b, more than 90% of ^125^I was found to remain on the PEG-AuNPs for at least 72 h in PBS. In the case of the ^125^I-PEG-AuNP challenged by serum, more than 85% of ^125^I was still found to be retained on the nanoparticles after 72 h of incubation. The results of the *in vitro* stability assays indicate that the ^125^I-PEG-AuNPs possess sufficient radiochemical stability and are suitable for biological applications. RCS of the ^125^I-PEG-AuNPs can most likely be further improved by preoxidizing [^125^I]NaI before adding it to the PEG-AuNPs or by using charged coating agents instead of PEG.^54^

**Figure 4:**
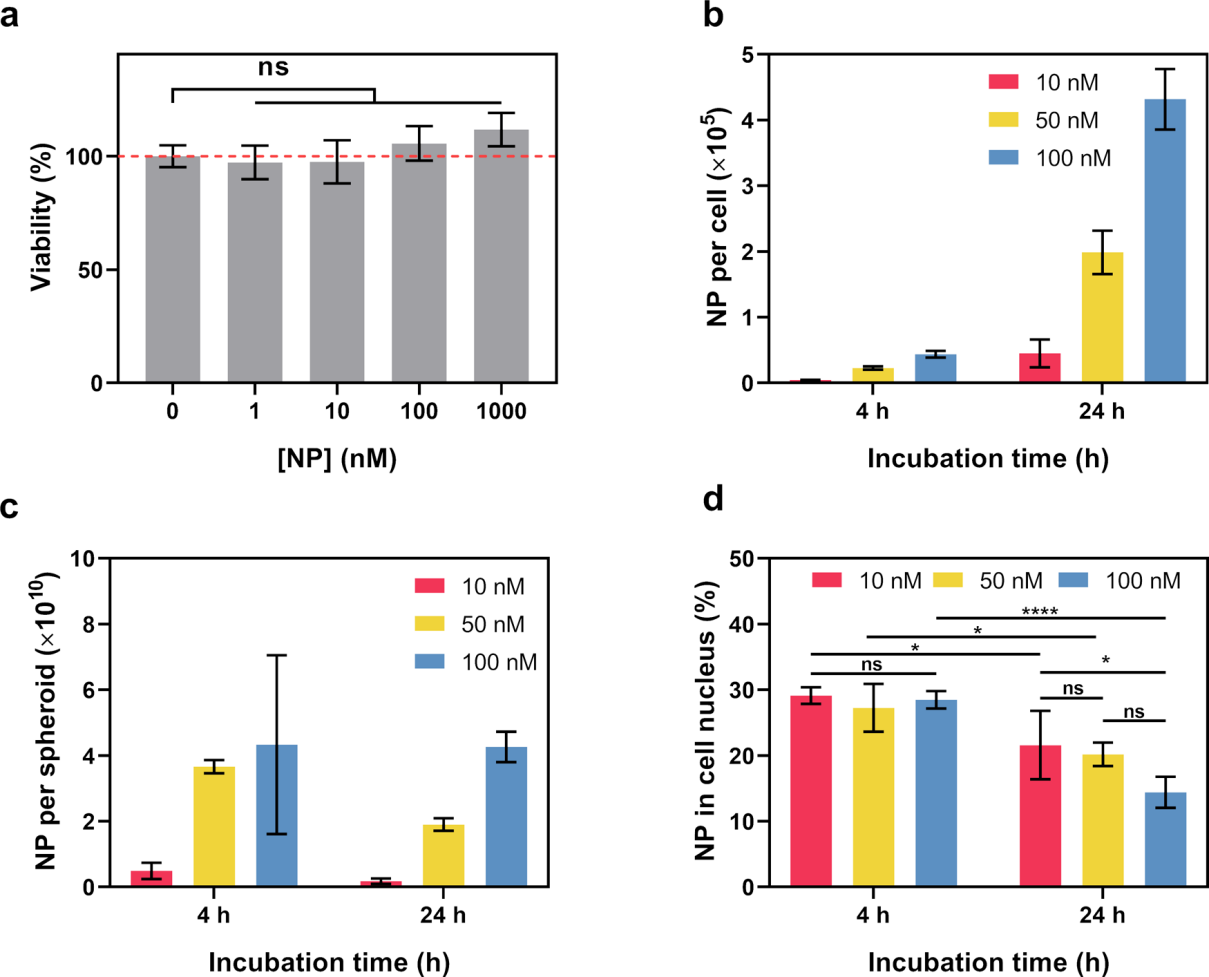
In vitro behavior of ^125^I-PEG-AuNPs: (a) viability of U87 cells treated with bare PEG-AuNPs at different concentrations, *n* = 5; (b) uptake of ^125^I-PEG-AuNPs in U87 cell monolayers after 4 and 24 h incubation at 37 °C, data are shown as number of nanoparticles per single cell, *n* = 3; (c) uptake of ^125^I-PEG-AuNPs in U87 cell spheroids after 4 and 24 h incubation at 37 °C, data are shown as number of nanoparticles per spheroid, *n* = 3; (d) subcellular distribution of ^125^I-PEG-AuNPs in U87 cell monolayers after 4 and 24 h incubation, data are shown as the percentage of nanoparticles present in the cell nucleus from all internalized nanoparticles, *n* = 3. *P* values: ns, *p* > 0.05, *p ≤ 0.05, ****p ≤ 0.0001.

To check the biocompatibility of the PEG-AuNPs, U87 cells were incubated with bare PEG-AuNPs for 24 h with concentrations ranging from 1 nM to 1 μM. The viability of the cells was then determined by the CCK-8 assay. As shown in Figure 4a, no significant difference on the cell viability was observed between the PEG-AuNPs treated groups and the control group, clearly indicating that the PEG-AuNPs are nontoxic to U87 cells even at a concentration as high as 1 μM. It needs to be mentioned that the cell proliferation seemed to be promoted at higher PEG-AuNP concentrations although the difference is not significant. Similar trends have been previously reported in the literature.^55−57^ As the viability results from these groups were not significantly different from those of the control group, the bare PEG-AuNPs were not expected to interfere with the follow-up cytotoxicity studies. The mechanism behind this phenomenon requires further investigation but is beyond the scope of this study.

The uptake of the PEG-AuNPs was then investigated in the U87 cell monolayers. The uptake of bare PEG-AuNPs was first studied by silver staining (Figure S5). It can be seen from the silver staining images that the PEG-AuNPs was either internalized into U87 cells or it was attached to the cell membrane. Next, ^125^I-PEG-AuNPs having 37 kBq ^125^I were mixed with nonradioactive PEG-AuNPs to achieve a final NP concentration of 10, 50, and 100 nM. The U87 cells were then incubated with the PEG-AuNPs for 4 or 24 h at 37 °C. The cell uptake of the PEG-AuNPs was found to be dependent on the concentration of the nanoparticles and the incubation time as shown in Figure 4b. By extending the incubation time from 4 to 24 h, the number of internalized nanoparticles increased by nearly 10 times for all tested concentrations. Furthermore, increasing the concentration of PEG-AuNPs from 10 to 100 nM resulted in an approximately 10 times higher uptake at 24 h. The highest cell uptake was achieved with 100 nM PEG-AuNPs and an incubation time of 24 h, reaching on average 4.3 × 10^5^ NPs internalized per cell. The subcellular distribution assay showed that approximately 15 to 20% of the internalized PEG-AuNPs were found to accumulate at the cell nucleus after 24 h of incubation (Figure 4d), matching well with the results from the literature.^50,58^ The distribution of internalized ^125^I-PEG-AuNPs in the cell membrane and cytoplasm fractions can be found in Figure S7.

The uptake of the ^125^I-PEG-AuNPs in 3D U87 cell spheroids was also evaluated for 10, 50, and 100 nM ^125^I-PEG-AuNPs having 37 kBq ^125^I. Surprisingly, the uptake of the ^125^I-PEG-AuNPs in spheroids seems to be saturated after 4 h of incubation. At 24 h, the number of nanoparticles per spheroid in all groups was similar to, or even lower than, that found at 4 h (Figure 4c). Due to the small size and nontargeted nature of the ^125^I-PEG-AuNPs, passive uptake, i.e., diffusion is the dominant transport pathway of the ^125^I-PEG-AuNPs inside the spheroids. Nanoparticles taken up by spheroids have been reported to mostly localize in the interstitial space instead of being internalized inside the cells.^59^ Based on the results from our 3D spheroids uptake experiments, we assume that the intravasation and extravasation of nanoparticles reached a balance around 4 h of incubation, thus leading to similar uptake at 4 and 24 h. However, higher spheroid uptake at 24 h in comparison to 3 h has been reported in the literature using negatively charged gold nanoparticles with a diameter of 2 nm and coated by a small molecule (tiopronin).^49^ Thus, the PEG coating might hinder the penetration of the ^125^I-PEG-AuNPs inside the spheroids, probably due to the steric hindrance between the nanoparticles and the spheroid extracellular matrix (ECM).^60−62^

**Figure 5:**
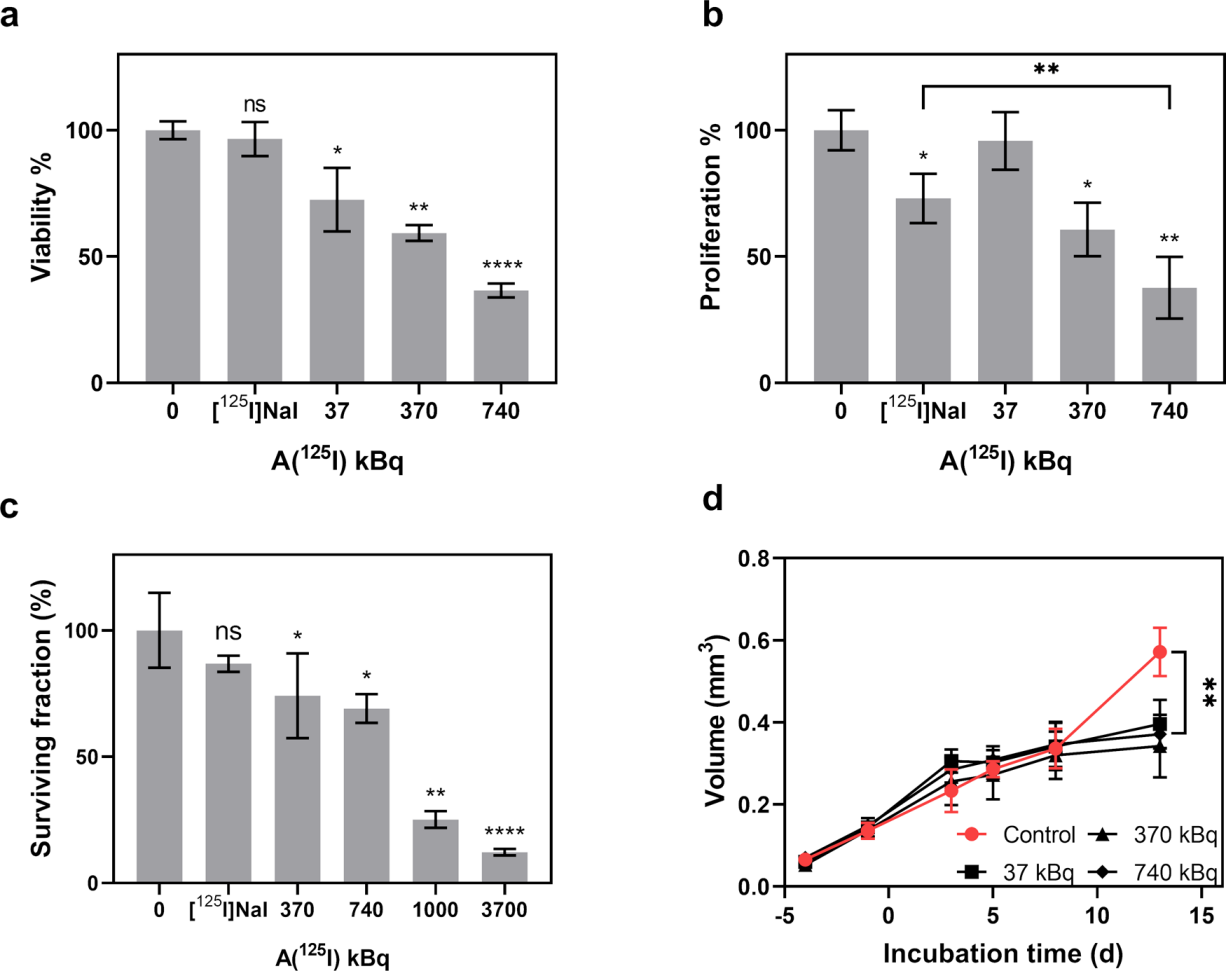
In vitro tumor-killing efficiency of ^125^I-PEG-AuNPs or [^125^I]NaI with different ^125^I activity determined by (a) viability assay 24 h after removal of activity. The specific activity of the ^125^I-PEG-AuNPs in each sample was 13.3 kBq/nM. The activity of [^125^I]NaI was 740 kBq, *n* = 4; (b) DNA proliferation assay 24 h after removal of activity. The activity of [^125^I]NaI is 740 kBq, *n* = 4; (c) colony formation assay. The specific activity of the ^125^I-PEG-AuNPs in each sample was 26.3 kBq/nM. The activity of [^125^I]NaI is 3.7 MBq, *n* = 3; (d) 3D spheroid growth inhibition assay. The spheroids were treated by ^125^I-PEG-AuNPs with 37, 370, or 740 kBq of ^125^I for 24 h. The specific activity of the ^125^I-PEG-AuNPs in each sample was 13.3 kBq/nM. The size change of the spheroids was monitored for another 13 days after the removal of activity. Nontreated spheroids were used as control, *n* = 4. *P* values: ns, *p* > 0.05, *p ≤ 0.05, **p ≤ 0.01, ***p ≤ 0.001, ****p ≤ 0.0001.

Motivated by the accumulation of ^125^I-PEG-AuNPs in the cell nucleus, the tumor-killing efficiency of the ^125^I-PEG-AuNPs was evaluated by using both 2D and 3D *in vitro* cell models. U87 monolayer cells were treated by ^125^I-PEG-AuNPs with ^125^I activity ranging from 37 to 740 kBq and an exposure time of 24 h. The viability of the cells was measured 24 h later after the removal of unbounded ^125^I-PEG-AuNPs. As shown in Figure 5a, a significant decrease in cell viability was detected after treatment with the ^125^I-PEG-AuNPs. The cell viability was reduced to only 36% when using 740 kBq of ^125^I. Moreover, the cells exhibited more than 95% viability after the treatment with 740 kBq of [^125^I]NaI, suggesting that the PEG-AuNPs played a vital role in cell killing.

After the measurement of cell viability, the DNA content of each sample was quantified using a commercial dsDNA quantification kit. By comparing the DNA content of the ^125^I-PEG-AuNPs treated groups with the control groups, the antiproliferation effect of the ^125^I-PEG-AuNPs on U87 could be determined.^63^ As shown in Figure 5b, a similar trend as found for the cell viability was observed for the proliferation efficiency of ^125^I-PEG-AuNPs treated cells. The highest cell proliferate inhibition was achieved by 740 kBq ^125^I-PEG-AuNPs where the proliferation efficiency was only 38%. The same ^125^I activity of [^125^I]NaI resulted in much less reduction of the cell proliferation efficiency (73%). The results of the DNA proliferation assay further supported the viability assay and clearly indicated that a high level of cell killing could be achieved with the ^125^I-PEG-AuNPs.

The viability and DNA content of the U87 cells were also measured 48 h after the removal of ^125^I-PEG-AuNPs. Reduction of cell viability and proliferation were again observed but were less pronounced compared to the 24 h results, probably due to repair of the inflicted DNA damage (Figure S8).

To further verify the high tumor-killing efficiency of the ^125^I-PEG-AuNPs, a colony formation assay was performed to directly determine the level of cell reproductive death. The U87 cells were first treated by ^125^I-PEG-AuNPs with the increasing activity of ^125^I ranging from 370 kBq to 3.7 MBq and then reseeded on 6-well plates for colony formation. The surviving fraction of each group was then calculated and is shown in Figure 5c. After being treated with 370 and 740 kBq ^125^I-PEG-AuNPs, the surviving fraction of U87 cells was reduced to ∼70%. Increasing the ^125^I from 370 kBq to 1 MBq resulted in a 3-fold decrease in surviving fraction, i.e., from 74 to 25%. When even higher ^125^I activity (3.7 MBq) was applied, the surviving fraction was reduced to only 12% compared with the untreated cells.

To better understand the radiation damage produced by the ^125^I-PEG-AuNPs, the absorbed dose of a single U87 cell at the time of execution of the viability assay and colony formation assay was calculated. For the U87 cells exposed to ^125^I-PEG-AuNPs containing 740 kBq of ^125^I during the viability assay (Figure 5a), a dose of 10.5 Gy per cell was calculated in 24 h. The longer irradiation time (14 days) and the higher ^125^I activity used in the colony formation assay led to even higher doses of 11.7 and 48.3 Gy per cell for the 1 and 3.7 MBq groups, respectively. As cell nucleus is the primary target of Auger therapy, the dose deposited in the cell nucleus was also calculated and found to be 14.0 Gy/cell, 13.5 Gy/cell, and 50.2 Gy/cell, respectively, for the three different treatments described above.

As the spheroid models could mimic the difference between the actual tumor and monolayer cell models, the tumor-killing efficiency of the ^125^I-PEG-AuNPs was also assessed in U87 cell spheroids.^64^ The size of the spheroids was monitored for up to 14 days after the treatment with ^125^I-PEG-AuNPs. As shown in Figure 5d, no difference between the volumes of the ^125^I-PEG-AuNPs treated spheroids and control spheroids was observed at early time points. On day 14, the growth of the treated spheroids seemed to be better controlled, while the untreated spheroids kept on growing. However, no influence of the administered ^125^I activity on the spheroid growth was observed. This somewhat low response is most likely due to passive internalization of the ^125^I-PEG-AuNPs, which might result in particles diffusing out of the spheroid. Despite the low uptake, the spheroids were continuously irradiated by ^125^I over the 14 days of incubation due to the long half-life of ^125^I and resulted in better controlled growth. To determine whether the spheroid growth inhibition is a result of the treatment with the ^125^I-PEG-AuNPs, spheroids were treated with 740 kBq [^125^I]NaI, showing no obvious decrease of spheroid size over 14 days of incubation (Figure S9).

In this work, we developed an ^125^I based radiopharmaceutical using ultrasmall gold nanoparticles as carriers and evaluated their tumor uptake and killing efficiency *in vitro*. Compared with antibodies or peptide-based carriers, the *in vitro* tumor uptake of the ^125^I-PEG-AuNPs is the modest. Possible reasons include the small size and the PEG coating on the AuNPs. The uptake mechanism of nanomaterials into cells is influenced by many factors including the size, surface charge, and surface modification of the nanoparticles.^65^ It has been experimentally shown that nanoparticles with a diameter of around 40 nm have the highest *in vitro* cell uptake while the uptake is lower for small nanoparticles (2–10 nm).^66^ The formation of a protein corona on the nanoparticles also plays an essential role in cell uptake. Previous research has proposed that higher uptake of 2 nm sized nanoparticles could be achieved at higher serum conditions.^67^ In the case of our PEG-AuNPs, the protein interaction is minimized due to the coating of PEG molecules, thus lowering the cell uptake.

Despite the modest uptake, the ^125^I-PEG-AuNPs were found to kill the tumor cells efficiently as observed from the *in vitro* experiments. Due to the high number of AEs emitted per decay of ^125^I (24.9 AEs per decay) and relatively high LET of these AEs, even a tiny amount of activity accumulated in the nucleus seemed to provide a sufficient radiation dose to induce damage to the DNA molecules (Figure S6). Moreover, the interaction between ^125^I and the gold surface also favors tumor cell killing. Gold nanoparticles have been widely applied as radiosensitizers in external beam radiation therapy (EBRT). As a high Z element, gold atoms can interact with photons or electrons and emit a high number of secondary photons and electrons which leads to higher tumor-killing efficiency.^68^ To verify the radiosensitizing effects of the PEG-AuNPs in this study, we also performed dose calculations based on simplified simulations using the same ^125^I activity and subcellular distribution but without the presence of gold. As shown in Table S1, the presence of PEG-AuNPs increased the radiation dose in a single U87 cell by approximately 30% when compared to only ^125^I. When comparing the dose deposited in the cell nucleus, the presence of PEG-AuNPs resulted in about two times higher dose than only ^125^I. These results clearly demonstrate the vital role of the PEG-AuNPs in radiosensitizing.

Although promising results were achieved, this work still has certain limitations for future clinical translation. First, the fraction of the internalized ^125^I-PEG-AuNPs in the nucleus was determined using a subcellular fractionation kit. Additional measurements, such as TEM imaging and confocal imaging, might provide more evidence for the accumulation of the ^125^I-PEG-AuNPs in the nucleus. Current work focused on the *in vitro* studies while the *in vivo* behavior of the ^125^I-PEG-AuNPs remains unknown. *In vivo* studies have to be performed to verify renal clearance and the tumor growth inhibition effect of the ^125^I-PEG-AuNPs. Considering the transport barriers experienced by injected nanoparticles before reaching the tumor cells, it might be challenging to deliver a high radiation dose of ^125^I-PEG-AuNPs to the cell nucleus under *in vivo* conditions.^69^ Surface functionalization by tumor-targeting agents such as PSMA inhibitors is expected to improve tumor uptake while maintaining the small dimensions of the nanoparticles.^70^

## 4 Conclusions

In this work, we explored a new type of radiopharmaceutical using AE emitters and ultrasmall nanoparticles. The high tumor-killing efficiency of the ^125^I-PEG-AuNPs was systematically studied by using various *in vitro* models. The high tumor-killing efficiency was attributed to accumulation of the nanoparticles in the cell nucleus, as well as the high yield of AEs originating from ^125^I. The results obtained from this work provide a new path for the application of AE emitters and hopefully offer new possibilities for cancer treatment.

## Supporting Information Available

Calculation of the number of internalized PEG-AuNPs per cell; normalized UV–Vis spectrum of PEG-AuNPs in PBS at different time points at 37 °C; ^125^I radiolabeling efficiency on PEG-AuNPs using [^125^I]NaI solution at pH of 12–14 or neutralized to pH 7; ^125^I radiolabeling efficiency on PEG-AuNPs with various ^125^I to NP ratios; typical iTLC radiochromatogram of ^125^I-PEG-AuNPs right after radiolabeling; silver staining of U87 monolayer cells treated by PEG-AuNPs; uptake of ^125^I-PEG-AuNPs in U87 cell monolayers and spheroids as percentage of initially added ^125^I activity; subcellular distribution of ^125^I-PEG-AuNPs in the other parts of U87 cells; *in vitro* tumor-killing efficiency of ^125^I-PEG-AuNPs determined at 48 h after the removal of activity; 3D spheroid growth inhibition assay of 740 kBq [^125^I]NaI treated U87 spheroids; and parameters used in the dosimetry calculations and the calculated absorbed dose in the cell nucleus or the whole cell (PDF)
