# Supplementary material for: Ultrasmall Gold Nanoparticles Radiolabeled with Iodine-125 as Potential New Radiopharmaceutical
Source: ACS Appl Bio Mater. 2024 Feb 7;7(2):1240–9. doi: 10.1021/acsabm.3c01158 (PMC10880057; doi:10.1021/acsabm.3c01158)
Supplement: Supplementary file 1 — mt3c01158_si_001.pdf [file mt3c01158_si_001.pdf]

Supporting Information

Ultra-small gold nanoparticles radiolabeled with Iodine-125 as potential new radiopharmaceutical

Runze Wang[1], Huanhuan Liu[2], Bas Antal[1], Hubert Th. Wolterbeek[1] & Antonia G. Denkova[1*]

[1]Applied Radiation and Isotopes, Department of Radiation Science and Technology, Faculty of Applied Sciences, Delft University of Technology, Mekelweg 15, 2629 JB, Delft, the Netherlands

[2]Department of Medical Imaging, Henan Provincial People's Hospital & the People's Hospital of Zhengzhou University, Zhengzhou, 450003 P. R. China

[*]Corresponding author: Antonia Denkova

E-mail addresses: A.G.Denkova@tudelft.nl

Calculation of the number of internalized PEG-AuNPs per cell

According to the ^{125}I counts measurement of the detached cells and the washing solutions, the percentage of initially added ^{125}I that being taken by the cells (Y%) can be calculated with the following equation:

$$Y\% = \frac{Counts(cells)}{Counts(washing\ solution) + Counts(cells)} \times 100\%$$

As the ^{125}I-PEG-AuNPs and the non-radioactive PEG-AuNPs were homogenously mixed before the addition to cells, the ^{125}I-PEG-AuNPs could be considered as a tracer of all PEG-AuNPs. Thus, the percentage of internalized ^{125}I activity was equal to the percentage of internalized PEG-AuNPs.

The number (N) of initially added PEG-AuNPs (as a sum of radioactive and non-radioactive) can be calculated using the concentrations of PEG-AuNPs (c, in nM) and the volume of stock solution that were added to the cells (v, in ml).

$$N = c \times 10^{-9} \times v \times 10^{-3} \times 6.02 \times 10^{23}$$

With the number of cells (M) per well, the number of internalized PEG-AuNPs per cell was hereby calculated using the following equation:

$$NPs/cell = \frac{N \times Y\%}{M}$$

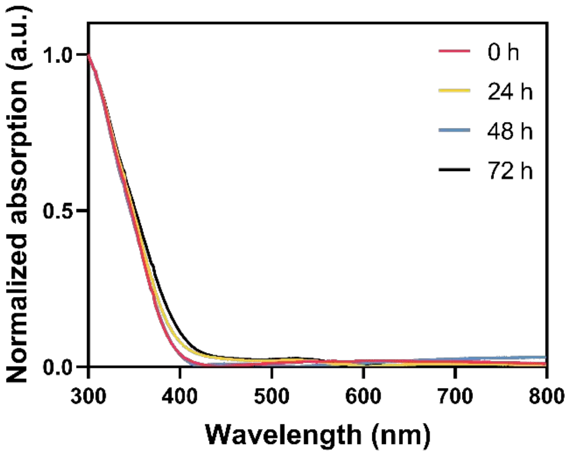

Figure S1. Normalized UV-vis spectrum of PEG-AuNPs in PBS at different time points at 37 ℃.

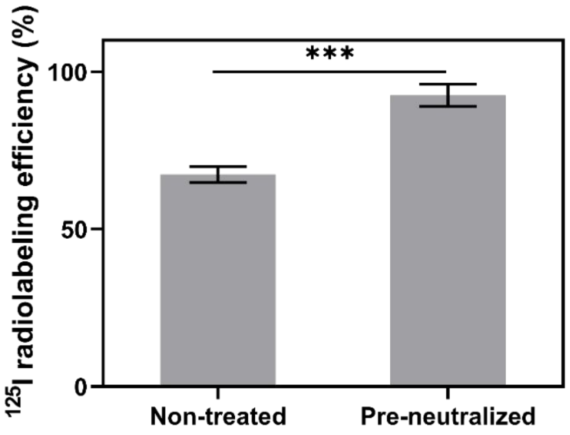

Figure S2. ^{125}I radiolabeling efficiency on PEG-AuNPs using [^{125}I]NaI solution at pH of 12~14 or neutralized to pH 7. The ^{125}I to NP ratio was set to 0.1 for all samples, n=3.

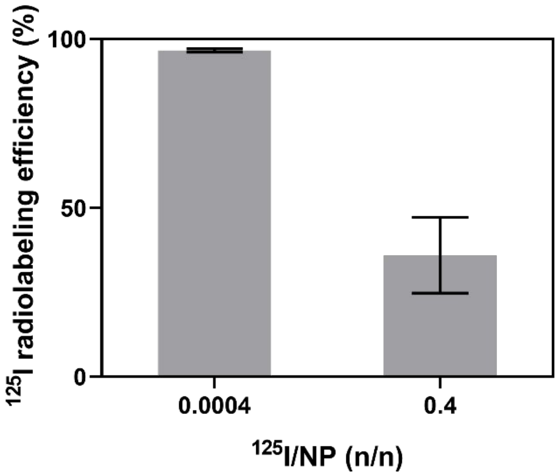

Figure S3. ^{125}I radiolabeling efficiency on PEG-AuNPs with various ^{125}I to NP ratios. [^{125}I]NaI solution was neutralized before the radiolabeling of all samples, n=3.

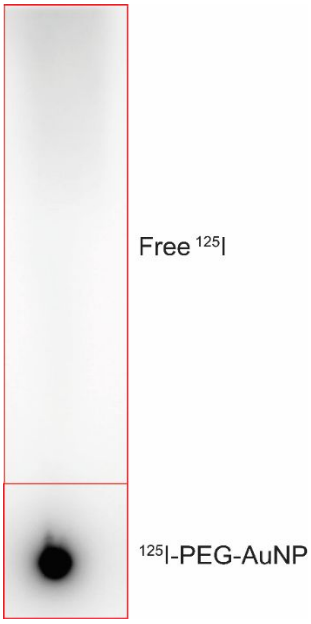

Figure S4. Typical iTLC radiochromatogram of ^{125}I-PEG-AuNPs right after radiolabeling. The ^{125}I radiolabeled on the PEG-AuNPs remained at the origin while the free $^{125}I^-$ moved along with the mobile phase (mobile phase: acetonitrile:water=1:3)

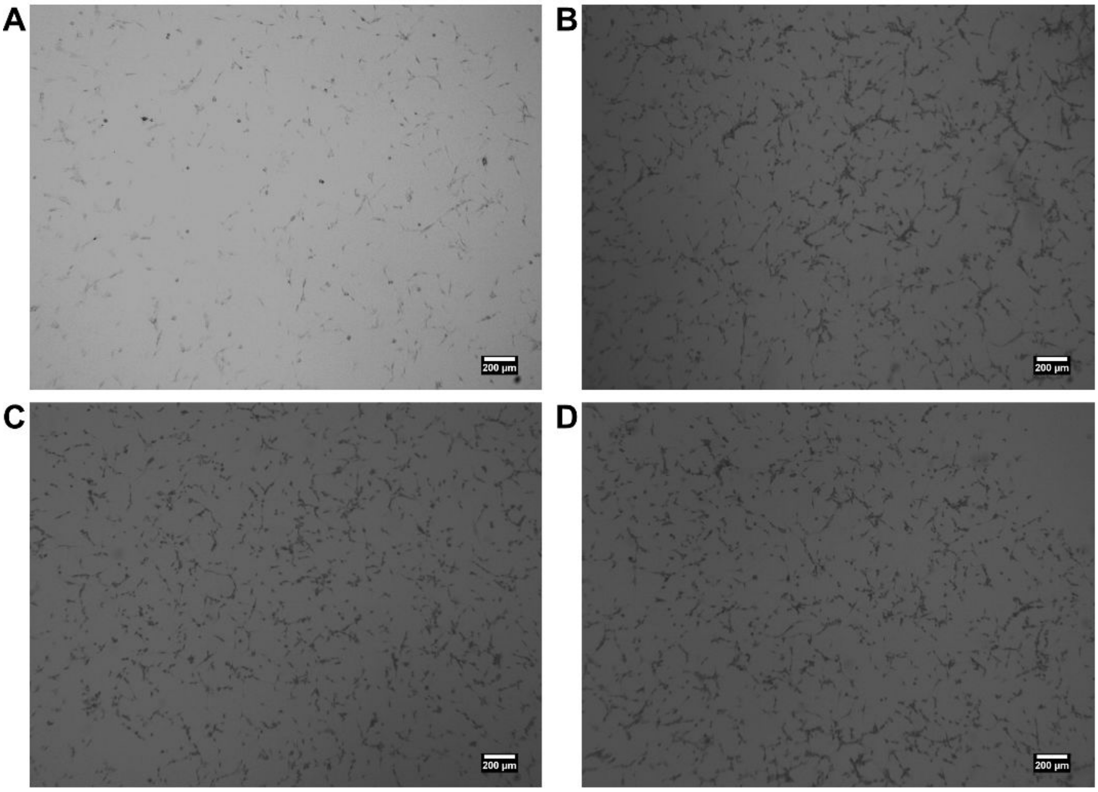

Figure S5. Silver staining of U87 monolayer cells treated by a) 0 nM; b) 1 nM; c) 50 nM and d) 100 nM bare PEG-AuNPs for 24 h. The scale bar is 200 µm. The increase contrast of the cells indicates the uptake of nanoparticles.

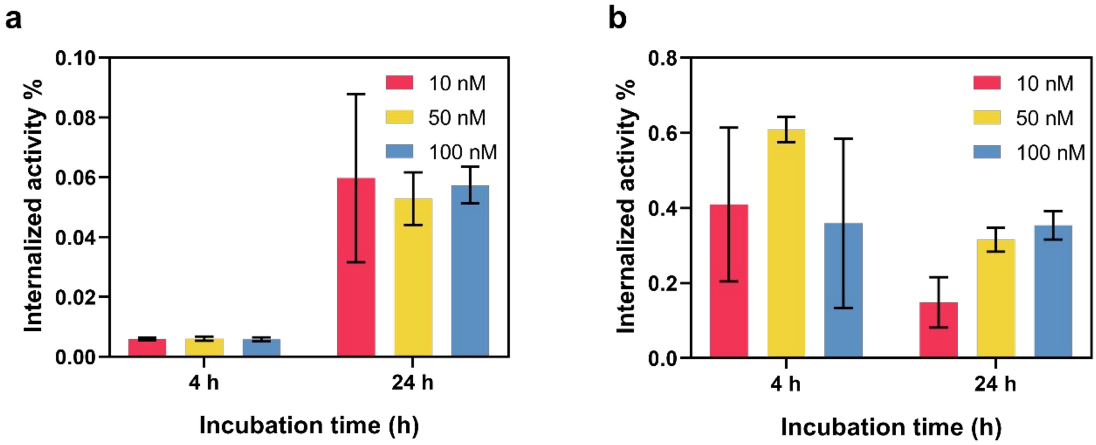

Figure S6. a) Uptake of ^{125}I-PEG-AuNPs in U87 cell monolayers after 4 and 24 h incubation at 37 °C, data is shown in percentage of initially added ^{125}I activity, n=3; b) uptake of ^{125}I-PEG-AuNPs in U87 cell spheroids after 4 and 24 h incubation at 37 °C, data is shown in percentage of initially added ^{125}I activity, n=3.

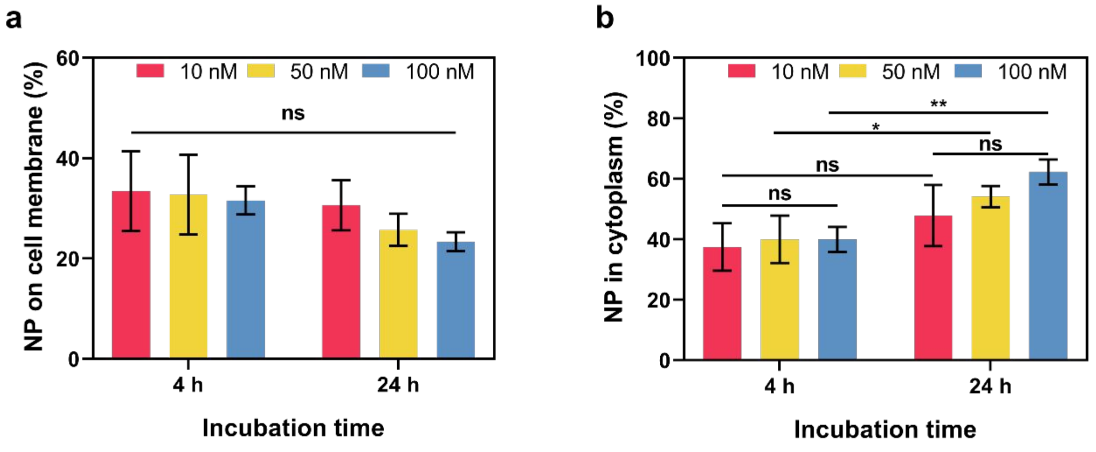

Figure S7. Sub-cellular distribution of ^{125}I-PEG-AuNPs on the a) cell membrane and b) in the cell cytoplasm of U87 cell monolayers after 4 and 24 h incubation, data is shown as percentage of all internalized nanoparticles, n=3.

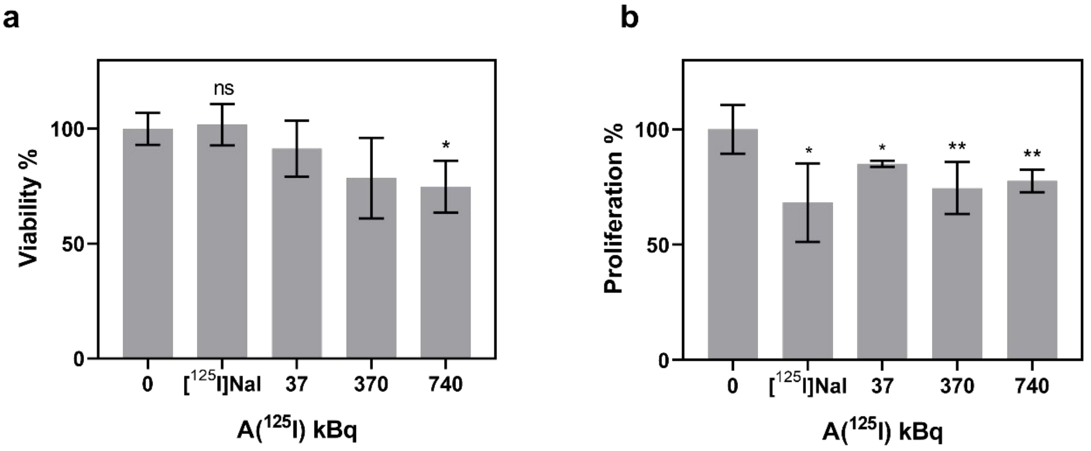

Figure S8. *In vitro* tumor killing efficiency of ^{125}I-PEG-AuNPs with different ^{125}I activity or 740 kBq [^{125}I]NaI determined by a) viability assay 48 h after removal of activity, n=4; b) DNA proliferation assay 48 h after removal of activity, n=4.

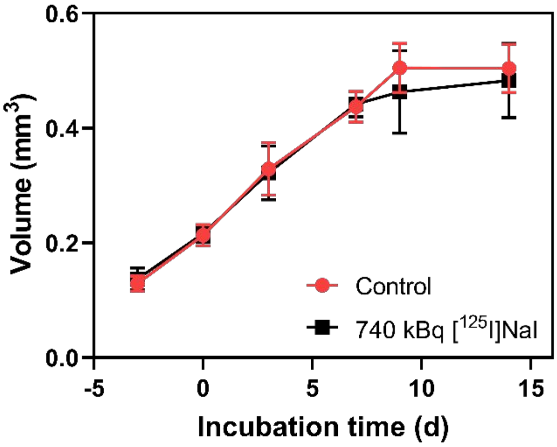

Figure S9. 3D spheroid growth inhibition assay of 740 kBq [^{125}I]NaI treated U87 spheroids. Non-treated spheroids were used as control, n=4.

Table S1. Parameters used in the dosimetry calculations and the calculated absorbed dose in the cell nucleus or the whole cell.

Sample Nr.	1	2	3	4	5	6
Corresponding cytotoxicity assay sample	Viability assay, 740 kBq		Colony formation assay, 1 MBq		Colony formation assay, 3.7 MBq	
A(^{125}I) per cell/Bq	0.078	0.078	0.007	0.007	0.027	0.027
A(^{125}I) in cell nucleus/Bq	0.016	0.016	0.001	0.001	0.004	0.004
A(^{125}I) on cell membrane/Bq	0.042	0.042	0.004	0.004	0.017	0.017
A(^{125}I) in cytoplasm/Bq	0.020	0.020	0.002	0.002	0.006	0.006
m(Au) per cell/fg	0	22.04	0	13.78	0	29.91
m(Au) in cell nucleus/fg	0	4.45	0	2.78	0	4.31
m(Au) on cell membrane/fg	0	11.93	0	7.46	0	18.61
m(Au) in cytoplasm/fg	0	5.66	0	3.54	0	6.98
Irradiation time/h	24	24	336	336	336	336
Absorbed dose in single cell/Gy	7.839	10.517	9.269	11.688	37.347	48.319
Dose enhancement factor in presence of Au	1.342		1.261		1.294	
Absorbed dose in cell nucleus/Gy	6.197	13.974	7.328	13.520	22.987	50.182
Dose enhancement factor in presence of Au	2.255		1.845		2.183	
